# Gossypol Broadly Inhibits Coronaviruses by Targeting RNA‐Dependent RNA Polymerases

**DOI:** 10.1002/advs.202203499

**Published:** 2022-10-20

**Authors:** Wenjing Wang, Wenkang Li, Zhiyuan Wen, Chong Wang, Weilong Liu, Yufang Zhang, Juncheng Liu, Tianze Ding, Lei Shuai, Gongxun Zhong, Zhigao Bu, Lingbo Qu, Maozhi Ren, Fuguang Li

**Affiliations:** ^1^ Zhengzhou Research Base State Key Laboratory of Cotton Biology School of Agricultural Sciences Zhengzhou University Zhengzhou Henan 450001 P. R. China; ^2^ State Key Laboratory of Veterinary Biotechnology Harbin Veterinary Research Institute Chinese Academy of Agricultural Sciences Harbin Heilongjiang 150069 P. R. China; ^3^ Institute of Cotton Research Chinese Academy of Agricultural Sciences Anyang Henan 455000 P. R. China; ^4^ Institute of Urban Agriculture Chinese Academy of Agricultural Sciences Chengdu Sichuan 610213 P. R. China; ^5^ Hainan Yazhou Bay Seed Laboratory Sanya Hainan 572025 P. R. China; ^6^ Institute for Hepatology National Clinical Research Center for Infectious Disease Shenzhen Third People's Hospital The Second Affiliated Hospital School of Medicine Southern University of Science and Technology Shenzhen Guangdong 518112 P. R. China

**Keywords:** coronavirus, gossypol, natural product, RNA‐dependent RNA polymerase, SARS‐CoV‐2

## Abstract

Outbreaks of coronaviruses (CoVs), especially severe acute respiratory syndrome coronavirus 2 (SARS‐CoV‐2), have posed serious threats to humans and animals, which urgently calls for effective broad‐spectrum antivirals. RNA‐dependent RNA polymerase (RdRp) plays an essential role in viral RNA synthesis and is an ideal pan‐coronaviral therapeutic target. Herein, based on cryo‐electron microscopy and biochemical approaches, gossypol (GOS) is identified from 881 natural products to directly block SARS‐CoV‐2 RdRp, thus inhibiting SARS‐CoV‐2 replication in both cellular and mouse infection models. GOS also acts as a potent inhibitor against the SARS‐CoV‐2 variant of concern (VOC) and exerts same inhibitory effects toward mutated RdRps of VOCs as the RdRp of the original SARS‐CoV‐2. Moreover, that the RdRp inhibitor GOS has broad‐spectrum anti‐coronavirus activity against alphacoronaviruses (porcine epidemic diarrhea virus and swine acute diarrhea syndrome coronavirus), betacoronaviruses (SARS‐CoV‐2), gammacoronaviruses (avian infectious bronchitis virus), and deltacoronaviruses (porcine deltacoronavirus) is showed. The findings demonstrate that GOS may serve as a promising lead compound for combating the ongoing COVID‐19 pandemic and other coronavirus outbreaks.

## Graphical abstract

Gossypol is identified as an effective inhibitor against SARS‐CoV‐2 RNA‐dependent RNA polymerases. It exhibits excellent antiviral activity against SARS‐CoV‐2 and its variants of concern as well as all examined viruses from the four main phylogenetic branches of coronaviruses, which makes gossypol a novel broad‐spectrum anti‐coronavirus inhibitor that offers a potential treatment option for current COVID‐19 and future coronavirus infections.

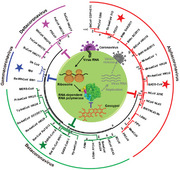

## 1 Introduction

The global health emergency resulting from severe acute respiratory syndrome coronavirus 2 (SARS‐CoV‐2) and its variants of concern (VOCs) are causing tremendous social and economic disasters worldwide. The introduction of vaccines has greatly contributed to SARS‐CoV‐2 prevention. However, the high mutation rate of SARS‐CoV‐2 and the spread of VOCs pose enormous challenges for the development of vaccines.^[^ ^1^ ^]^ Therefore, it is important to develop broad‐spectrum inhibitors for various SARS‐CoV‐2 variants. SARS‐CoV‐2 belongs to a broad family of viruses known as *Coronaviridae*, which causes serious damage to humans and animals. Coronaviruses (CoVs) have four main phylogenetic branches based on their genetic sequences: alphacoronaviruses, betacoronaviruses, gammacoronaviruses, and deltacoronaviruses. In addition to SARS‐CoV‐2, two other betacoronaviruses, severe acute respiratory syndrome coronavirus (SARS‐CoV) and Middle East respiratory syndrome coronavirus (MERS‐CoV), have caused severe epidemics or pandemics in humans over the past two decades. More seriously, some other coronaviruses—which are constantly evolving, breaking host species barriers, and expanding their host range—can spread among a wide variety of animal species with high infectivity and mortality,^[^ ^2^, ^3^ ^]^ thereby bringing a constant threat to the world. Recently, three coronaviruses including two canine alphacoronaviruses^[^ ^4^, ^5^ ^]^ and one porcine deltacoronavirus^[^ ^6^ ^]^ have been reported to infect humans. In February 2022, scientists found that raccoon dogs, civets, porcupines and other wild animals could carry a variety of coronaviruses, some of which are at risk of cross‐species transmission.^[^ ^7^ ^]^ Therefore, a highly effective antiviral with broad‐spectrum coronavirus coverage would facilitate the control of existing and emerging coronavirus diseases.

Many host and viral proteins are involved in coronavirus replication. The viruses enter host cells through cell receptors, release their viral genome into the cell cytoplasm,^[^ ^8^, ^9^, ^10^ ^]^ and then translate to large polyproteins in the cell. The polyproteins are cleaved into several non‐structural proteins (nsps) by their own proteases such as papain like protease (PLpro)^[^ ^11^ ^]^ and main protease (Mpro).^[^ ^12^ ^]^ Several nsps coalesce to form a multiprotein replicase‐transcriptase complex (RTC) with RNA‐dependent RNA polymerase (RdRp) as the core.^[^ ^13^ ^]^ RdRp directly mediates the replication and transcription of viral RNA (vRNA), which plays a critical role in viral amplification. Moreover, RdRps are highly conserved in single‐stranded RNA viruses (ssRNA viruses),^[^ ^14^, ^15^, ^16^, ^17^ ^]^ especially CoVs,^[^ ^18^ ^]^ which provide promising targets for the discovery of broad‐spectrum anti‐CoV drugs. Generally, RdRp inhibitors fall into two main categories according to their mode of action and structure—nucleoside inhibitors (NIs) that act at the substrate site and non‐nucleoside inhibitors (NNIs) that interact with active or allosteric sites.^[^ ^19^ ^]^ Currently, several NIs, including remdesivir,^[^ ^20^ ^]^ molnupiravir (EIDD‐2801),^[^ ^21^ ^]^ favipiravir,^[^ ^22^ ^]^ GS‐441524^[^ ^23^ ^]^ and ATV006,^[^ ^24^ ^]^ have been found to be effective against SARS‐CoV‐2, making great contributions to fight the COVID‐19 pandemic. However, considering factors such as therapeutic impacts, adverse effects, feasible synthesis and cost‐effectiveness, more well‐developed inhibitors are still required.^[^ ^25^ ^]^ Phytochemical bioactives, with properties of high diversity, broad‐spectrum activity, overall safety and non‐cytotoxicity, are potential valuable candidates for anti‐CoV interventions.^[^ ^26^ ^]^ Several phytonutrients have been developed as RdRp NNIs for COVID‐19 interventions, such as baicalein,^[^ ^27^ ^]^ silibinin^[^ ^28^ ^]^ and corilagin (RAI‐S‐37),^[^ ^29^ ^]^ providing a potential option for COVID‐19 management. Although the above inhibitors have made a great contribution to SARS‐CoV‐2 prevention and control, their ability to inhibit other coronaviruses needs to be further explored.

Plants produce a wide variety of secondary metabolites that have evolved for a particular biological function, acting as a reservoir for drug discovery.^[^ ^30^ ^]^ Compared to other plants, *Gossypium* spp. (cotton plant) display strong resistance to ssRNA viruses. According to statistics provided by the International Committee on Taxonomy of Viruses (ICTV) and the National Center for Biotechnology Information (NCBI) virus database, many ssRNA viruses are known to infect major crops, including maize, rice, wheat, potato and tomato (Figure S1 and Table S1, Supporting Information); however, ssRNA virus infection is very rare in *Gossypium* plants. Their high resistance to ssRNA viruses may result from active plant‐derived natural products, such as the phenolic aldehyde compound gossypol (GOS), and flavonols such as gossypetin (GOP) and gossypin,^[^ ^31^, ^32^, ^33^, ^34^, ^35^, ^36^, ^37^ ^]^ which provide valuable resources for antiviral drug discovery.

**Scheme 1:**
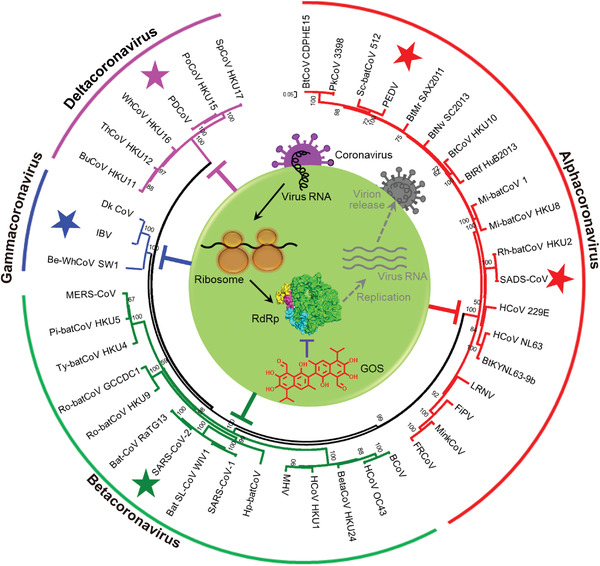
Schematic illustration of gossypol acting as a pan‐coronavirus inhibitor by blocking their highly conserved RNA‐dependent RNA polymerases.

This study aimed to discover cotton natural products that can inhibit coronaviruses over a broad spectrum. First, based on virtual screening and biochemical approaches, we performed screening using SARS‐CoV‐2 RdRp as the target and cotton natural products as the antiviral library. Then, antiviral effects of obtained SARS‐CoV‐2 RdRp inhibitors were assessed using in vitro and in vivo anti‐SARS‐CoV‐2 assays. Finally, based on the inhibitory mechanism of GOS against SARS‐CoV‐2 RdRp obtained by cryo‐electron microscopy (cryo‐EM), we speculated and verified the ability of GOS to inhibit SARS‐CoV‐2 VOCs and other coronaviruses, making it a potentially important tool against future pandemics (**Scheme** **1**).

## 2 Results

### 2.1 RdRp of SARS‐CoV‐2 Is Inhibited by GOS In Vitro

Given that plant natural products may play a role in chemical defense against viruses and should serve as a large pool for screening previously undescribed antiviral agents,^[^ ^38^ ^]^ we performed large‐scale computational screening to identify candidate inhibitors against SARS‐CoV‐2 RdRp from a library containing 881 cotton natural products (Table S2, Supporting Information). Baicalein, a known plant‐derived non‐nucleoside RdRp inhibitor,^[^ ^27^ ^]^ was used as a positive control. As a result, 50 anti‐SARS‐CoV‐2 RdRp candidates, including GOS, GOP, gossypin, myricitrin, astragalin, astilbin and kaempferitrin, each with known antiviral properties,^[^ ^39^, ^40^, ^41^, ^42^, ^43^ ^]^ had predicted energies with the active region of RdRp less than that of the positive control (Table S2, Supporting Information).

**Figure 1:**
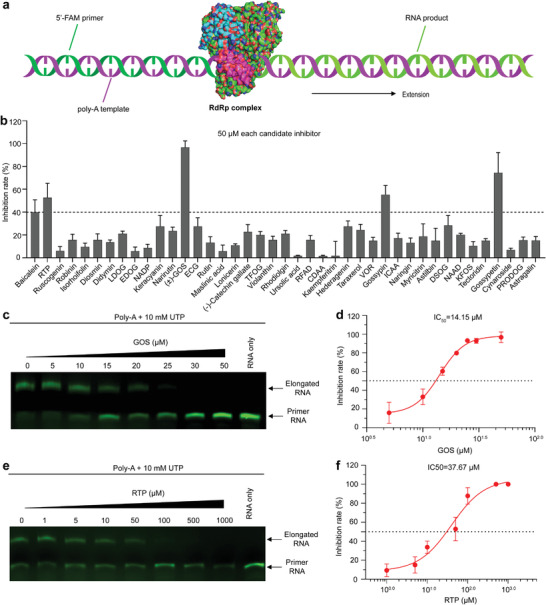
Inhibition of SARS‐CoV‐2 RdRp by GOS. a) Schematic diagram of the RNA extension assay based on SARS‐CoV‐2 RdRp. b) The screening of candidate RdRp inhibitor compounds from cotton plant using RNA extension assay based on SARS‐CoV‐2 RdRp. The working concentration of the inhibitors was 50 µm. LDOG: Luteolin‐7,3″‐di‐O‐glucoside; ECG: Epicatechin gallate; TFOG: Taxifolin‐3″‐O‐glucoside; RFAD: Riboflavin 5″‐Adenosine Diphosphate; CDAA: Cyclic di‐3″,5″‐adenylic acid; VOR: Vitexin‐2‐O‐rhamnoside; ICAA: Isochlorogenic acid A; DSOG: Diosmetin‐7‐O‐glucoside; NAAD: Nicotinic acid adenine dinucleotide; KFOS: Kaempferol‐3‐O‐sambubioside; PRODOG: Pinoresinol‐4,4″‐O‐di‐O‐glucoside. c,e) Gel‐based assays of the elongation of partial RNA duplexes by the purified SARS‐COV‐2 RdRp, and inhibition of this elongation by GOS (c) and RTP (e). d,f), Inhibition curves for GOS (d) and RTP (f) activity, calculated with data from the fluorescence‐based assays. Data are presented as mean ± s.d. (*n* = 3).

We further characterized 39 candidate inhibitors that are available on the market using RNA extension assays based on SARS‐CoV‐2 RdRp (**Figure** **1**a). Baicalein was used as the positive control because of its effectiveness in the RNA extension assay (Figure S2, Supporting Information). Remdesivir triphosphate (RTP), a well‐known SARS‐CoV‐2 nucleoside RdRp inhibitor^[^ ^13^ ^]^ was also used to evaluate the potency of the inhibitors. As shown in Figure 1b, 50 µm GOS displayed the strongest inhibitory effect against SARS‐CoV‐2 RdRp, followed by GOP and gossypin. Notably, GOS, GOP, and gossypin displayed stronger inhibitory effects than baicalein and RTP. GOS, the strongest inhibitor of RdRp, was further tested using a gel‐based RdRp assay. With increasing concentrations of GOS, RNA production gradually decreased and was completely absent at 20–25 µm (Figure 1c), while 50–100 µm baicalein (Figure S2a, Supporting Information) or RTP (Figure 1e) was required to achieve the same effect. The derivatives of GOS, acetate GOS (GOSAc) and (−)‐GOS, were further examined and showed inhibitory effects against RdRp, similar to GOS (Figure S3a–d, Supporting Information). The half‐maximal inhibitory concentration (IC_50_s) of GOS, GOSAc, (−)‐GOS, baicalein and RTP toward RdRp given by fluorescence‐based assays were 14.15 (Figure 1d), 14.83 (Figure S3b, Supporting Information), 15.17 (Figure S3d, Supporting Information), 62.55 (Figure S2b, Supporting Information) and 37.67 µm (Figure 1f), respectively. Taken together, these results indicate that the enzymatic activity of SARS‐CoV‐2 RdRp was significantly inhibited by GOS and its derivatives in vitro.

### 2.2 GOS Suppresses the Replication of SARS‐CoV‐2 in Cellular Infection Model

**Figure 2:**
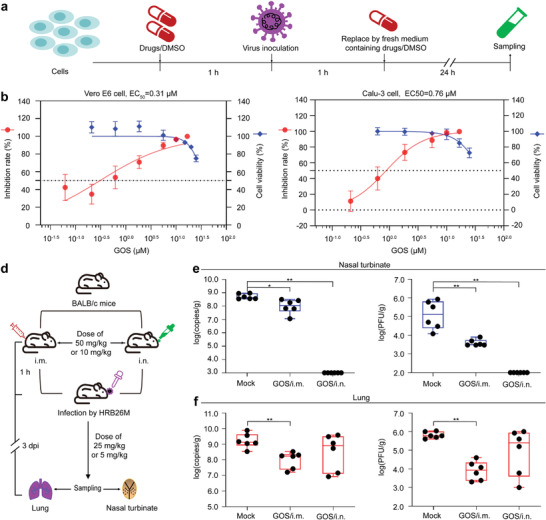
Cellular and animal infection models testing the anti‐SARS‐CoV‐2 effects of GOS. a) Experimental design for the cellular anti‐SARS‐CoV‐2 study. b,c) The inhibitory effects of GOS toward SARS‐CoV‐2 in Vero E6 cells (b) and Calu‐3 cells (c), and assessment of potential cytotoxicity. The *X*‐axis represents GOS working concentration. The left and right *Y*‐axes represent the inhibition rate and cell viability, respectively. Data are presented as mean ± s.d. (*n* = 3). d) Experimental design of anti‐SARS‐CoV‐2 study in a mice infection model. e,f) The viral RNA load (left figures in (e) and (f)) and infectious titers (right figures in (e) and (f)) in the nasal turbinates (e) and lungs (f) with high‐dose GOS. The viral RNA copies and infectious titers were detected by qPCR and viral titration. The i.m. represents for intramuscular treatment; i.n. represents for intranasal treatment. Data are presented as mean ± s.d. (*n* = 6). ** *p* < 0.01, * *p* < 0.05.

To evaluate the inhibitory effect of the candidate natural products from cotton plant on SARS‐CoV‐2 replication in vitro, an anti‐SARS‐CoV‐2 assay (**Figure** **2**a) was performed using Vero E6 cells^[^ ^44^ ^]^ with baicalein as a positive control. qPCR analysis of SARS‐CoV‐2 infected Vero E6 cells showed that GOS, GOSAc, (−)‐GOS, and GOP conferred stronger antiviral effects at 50 µm concentration than that of positive control (Table S3, Supporting Information). Further, we found the inhibitory effects of these compounds toward SARS‐CoV‐2 were dose‐dependent, with half‐maximal effective concentration (EC_50_) values of 0.31, 0.72, 0.84 and 33.22 µm, respectively (Figure 2b; Figures S3e,f and S4, Supporting Information). Additionally, there was no obvious cytotoxicity of the GOSs within the effective antiviral concentration range. The theoretical 50% cytotoxic concentrations (CC_50_s) and therapeutic indexes (TIs, CC_50_/EC_50_) for GOS, GOSAc and (−)‐GOS were 36.18, 44.51 and 35.43 µm, and 116.71, 61.82 and 42.17, respectively. Importantly, GOS still worked well in a human airway epithelial cell line Calu‐3 (EC_50_ = 0.76 µm, CC_50_ = 39.57 µm, TI = 52.07; Figure 2c), which was worth to further characterize its anti‐SARS‐CoV‐2 activity in vivo. Given that GOP caused cytotoxicity in the anti‐SARS‐CoV‐2 assay (Figure S4, Supporting Information), it was excluded from subsequent research.

### 2.3 GOS Inhibits the Replication of SARS‐CoV‐2 in Mouse Infection Model

We also assessed the anti‐SARS‐CoV‐2 activity of GOS in vivo using a previously reported mouse infection models.^[^ ^44^ ^]^ Female BALB/c mice were inoculated with mouse‐adapted SARS‐COV‐2/HRB26/human/2020/CHN (HRB26M), followed by daily treatment with GOS via the intramuscular (i.m.) and intranasal (i.n.) routes, respectively (Figure 2d). The vRNA load and infectious virus titer were measured in the nasal turbinates and lungs at 3 days post‐inoculation (dpi). In the high‐dose GOS‐treated group (Figure 2e,f), GOS treated i.n. significantly reduced HRB26M RNA load and HRB26M titer (*p* < 0.01) in the nasal turbinates compared to mock‐treated mice; GOS treated i.m. reduced the HRB26M load (*p <* 0.05) and titer (*p <* 0.01) in nasal turbinates, and significantly reduced these values (*p < 0.01*) in mouse lungs. In the low‐dose GOS‐treated group (Figure S5, Supporting Information), this dose scheme showed i.n. route was more effective than that of i.m. route in inhibiting SARS‐CoV‐2 replication in nasal turbinate, which showed the similar trend to the high‐dose group; However, neither i.n. nor i.m. route with low‐dose GOS showed obvious anti‐SARS‐CoV‐2 activity in lungs compared to that of mock group (*p >* 0.05), indicating higher i.m. dose is needed to obtain lung protection.

To further understand the better antiviral effect of GOS in mouse lungs via the i.m. route over the i.n. route, we analyzed the distributions of GOS in nasal turbinates and lungs under different administration routes with high‐dose GOS (Figure S6, Supporting Information). When treated i.n., the GOS in nasal turbinates was significantly higher than that in lungs at both 1 h post‐inoculation (hpi) and 3 dpi, which was in accordance with the better antiviral effect of GOS in the nasal turbinate than that in the lung. While treated i.m. resulted in a rapid accumulation of GOS in the lungs at 1 hpi, but not in the nasal turbinates; at 3 dpi, GOS concentration increased in both tissues with a significantly higher concentration in the lungs than that in the nasal turbinates, which explained the better anti‐SARS‐CoV‐2 activity in the lungs over the nasal turbinate when GOS was injected i.m. Therefore, discrepancies in GOS tissue distribution via the i.n. or i.m. route were highly consistent with the antiviral results.

### 2.4 Cryo‐EM Reveals that Two GOS Molecules Occupy the Active Site of SARS‐CoV‐2 RdRp

**Figure 3:**
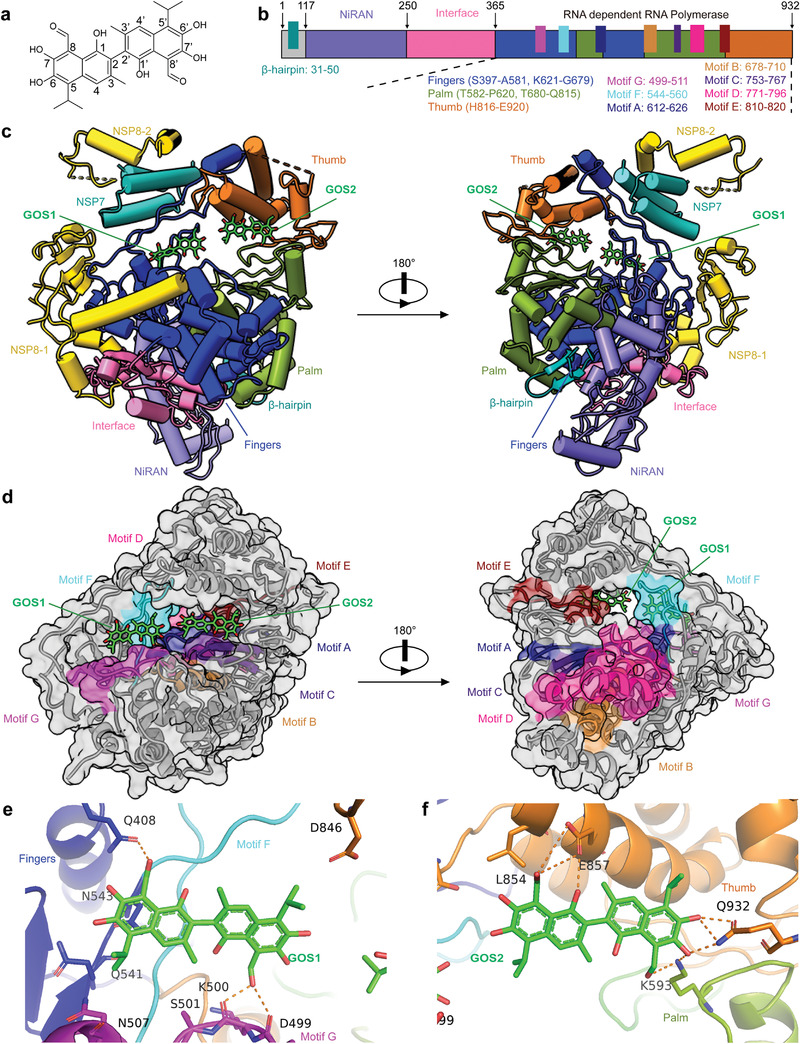
Cryo‐EM structure of the GOS‐bound RdRp complex. a) The structure of GOS. b) Schematic diagram for the components of NSP12. c) Two structure views of NSP12‐NSP7‐NSP8 in complex with GOS. GOS is colored green. d) Two surface views of the RdRp motifs A–G with bound GOS1 and GOS2. e,f) Zoom in views of the interactions between the SARS‐CoV‐2 RdRp and GOS molecules. The hydrogen bond is displayed as a blue dashed line.

To investigate how GOS (**Figure** **3**a) interacts with the SARS‐CoV‐2 RdRp complex (composed of NSP12, NSP7, and NSP8), we obtained cryo‐EM maps of the two types of complexes (Figure S7, Supporting Information). In one type of cryo‐EM map, the expected structural characteristics of the central cavity were observed (Figure S8a, Supporting Information). The other type of cryo‐EM map revealed a clear density in the central cavity of the RdRp complex (Figure S8b, Supporting Information), containing two GOS molecules (hereafter named GOS1 representing (−)‐GOS and GOS2 representing (+)‐GOS), one NSP12 core catalytic subunit (residues V31‐K50, Y69‐F102, P112‐L895, and N911‐Q932), one NSP7 cofactor subunit (residues K2‐S57), and two NSP8 cofactor subunits (NSP8‐1: T84‐L122 and M129‐I132, and NSP8‐2: D78‐A191) (Figure 3c). Consistent with a previously reported structure,^[^ ^45^ ^]^ the NSP12 we examined also contains five domains (NiRAN, Interface, Fingers, Palm, and Thumb) as well as seven highly conserved motifs (motifs A–G) that form the catalytic cavity for vRNA replication (Figure 3b). Two GOS molecules occupy the center of the central cavity and collectively reduce the size of the cavity opening (Figure 3c,d; Figure S8c, Supporting Information).

### 2.5 GOS Inhibits RdRp Activity by Occupying the Binding Site for the RNA Template and Primer

The SARS‐CoV‐2 RdRp–GOS structure showed that the GOS1 molecule binds to a groove composed of conserved G and F motifs of NSP12 (Figure 3e and Figure S8d, Supporting Information). The C‐8 active aldehyde group of GOS1 formed a hydrogen bond with residue Q408 of the NSP12 finger domain. The C‐8′ aldehyde group of GOS1 forms two hydrogen bonds with residues D499 and K500 of motif G. Additionally, van der Waals’ forces exist between GOS1 and NSP12 residues, including Q541, N543, N507 and D846. These collectively fix GOS1 in a relatively narrow cavity composed of motifs G and F. Another GOS molecule, GOS2, is close to GOS1 (Figure 3f and Figure S8e, Supporting Information). The GOS2 molecule's C‐8 aldehyde group and C1‐OH form three hydrogen bonds with residue E857 of the thumb domain of NSP12; the C‐8′ aldehyde group and C7′‐OH form two hydrogen bonds with residue K593 of palm domain; C7′‐OH and C6′‐OH groups form three hydrogen bonds with the final C‐terminal residue (Q932) of NSP12. These interactions help to stabilize the GOS2 molecule in a region formed by the thumb domain, palm domain, and C‐terminal residues of NSP12.

**Figure 4:**
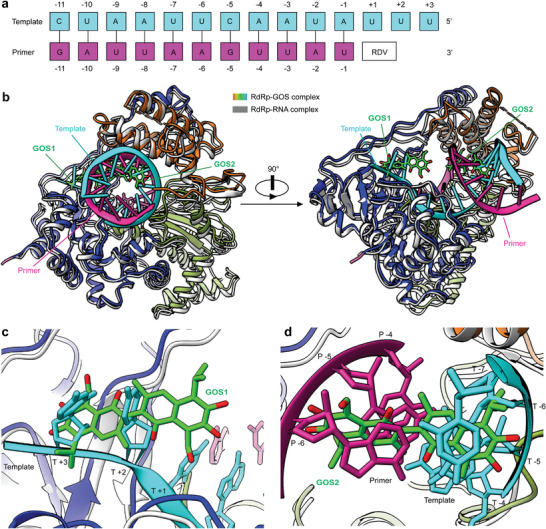
Structural comparisons of the GOS‐bound and RNA‐bound RdRp complexes. a) Definition of the base position of RTP and primer‐template RNA. b) Overall views of the SARS‐CoV‐2 RdRp–GOS complex overlapped with the RNA‐bond SARS‐CoV‐2 RdRp structure (PDB ID 7BV2). The RNA‐bond SARS‐CoV‐2 RdRp structure is shown in gray, the template RNA is cyan, and the primer RNA is purple. c) Zoom in view of GOS1 overlapped with RNA template strand from the RNA‐bond SARS‐CoV‐2 RdRp structure. d) Zoom in view of GOS2 overlapped with the RNA template and primer strands from the RNA‐bond SARS‐CoV‐2 RdRp structure.

Structural comparison of the RdRp–GOS complex with the RNA‐binding domain of the RdRp complex (PDB:7bv2) revealed a potential mechanism for the inhibition of RdRp by GOS (**Figure** **4**). The binding of GOS1 and GOS2 occupies sites known for primer–template duplex binding^[^ ^13^ ^]^ (Figure 4b). Specifically, if the base position of remdesivir (RDV) in 7bv2 is defined as position +1 (Figure 4a), the binding of GOS1 occupies space from positions +3 to +1 of the RNA template strands (Figure 4c). The binding of GOS2 sterically hinders the binding site of the primer–template duplex, which occupies: i) the space between positions −7 and −5 of the template strand; and ii) the space between positions −4 and −6 of the primer strand (Figure 4d). Therefore, the two GOS molecules blocked the binding of the RNA template–primer duplex to the active central cavity of NSP12, and this mechanism was further confirmed by the gel mobility shift assay (Figure S9, Supporting Information), in which the addition of GOS completely blocked the formation of the RdRp–RNA complex. The structural configuration of the GOS and RdRp complex consequentially inhibited the catalytic activity of RdRp and the replication of the SARS‐CoV‐2 RNA genome.

### 2.6 GOS Retains its Inhibition Capacity for RdRp Mutants and SARS‐CoV‐2 Variant

So far, most of the detected mutations in SARS‐CoV‐2 VOCs, including alpha, beta, gamma, delta and omicron, are mainly associated with structural proteins.^[^ ^46^ ^]^ However, non‐structural proteins such as RdRp are highly conserved and stable in mutant strains. In fact, only two amino acid mutations in RdRps have been identified in all the examined variants since 2019 (Figure S10a, Supporting Information). The RdRps of all VOCs, including omicron, carry the mutation P323L (RdRp^P323L^); the delta variant's RdRp has another mutation G671S (RdRp^P323L; G671S^). We tested the inhibitory activity of GOS against the RdRp^P323L^ and RdRp^P323L; G671S^ mutants. GOS displayed the same inhibitory effect on RdRp^P323L^ and RdRp^P323L; G671S^ as that on the original RdRp (Figure S10b–e, Supporting Information), suggesting that GOS can act as a pan‐inhibitor against SARS‐CoV‐2 variants. Assay with Vero E6 cells confirmed that the inhibition of GOS against SARS‐CoV‐2 is not altered in the variants of SARS‐CoV‐2 with EC_50_ of 0.23 µm and TI value of 157.30 toward the delta variant (Figure S10f, Supporting Information).

### 2.7 GOS Shows Broad Inhibitory Spectrum against Coronaviruses

**Figure 5:**
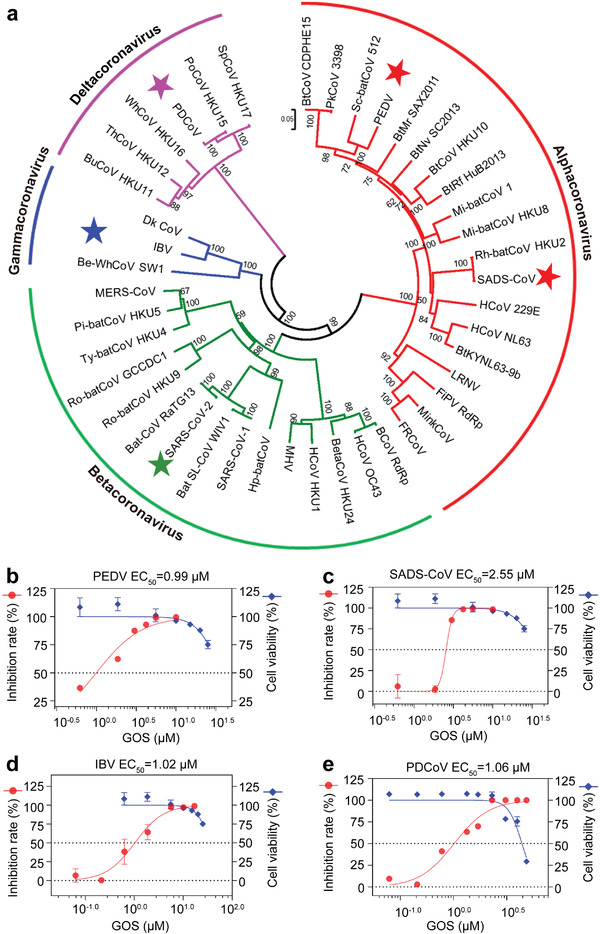
Broad spectrum inhibitory activity of GOS against coronaviruses. a) Phylogenetic trees created with RdRp amino acid sequences from all four CoV genogroups. b–e) The inhibitory effect of GOS on representative coronaviruses PEDV (b), SADS‐CoV (c), IBV (d), and PDCoV (e). Data are presented as mean ± s.d. (*n* = 3).

According to the multiple sequence alignment of coronavirus RdRps (Figure S11, Supporting Information), RdRps are known to be highly conserved. Therefore, we speculated that GOS may act as a broadband anti‐coronavirus drug by targeting the conserved RdRps. As shown in **Figure** **5**a, the four branches of the phylogenetic tree constructed using coronavirus RdRps correspond to the four genera of coronaviruses. In addition to SARS‐CoV‐2 from betacoronavirus, we randomly selected four representative coronaviruses to analyze the antiviral ability of GOS: porcine epidemic diarrhea virus (PEDV) and swine acute diarrhea syndrome coronavirus (SADS‐CoV) from alphacoronaviruses, infectious bronchitis virus (IBV) from gammacoronaviruses, and porcine deltacoronavirus (PDCoV) from deltacoronaviruses. The result showed that GOS exhibited significant inhibition against PEDV, SADS‐CoV, IBV and PDCOV with EC_50_s of 0.99, 2.55, 1.02 and 1.06 µm, respectively (Figure 5b,e). The anti‐CoV assays of PEDV, SADS‐CoV and IBV were performed using Vero E6 cells, and the TIs were 36.55, 14.19 and 35.47, respectively. The anti‐PDCoV assay was performed with swine testis (ST) cells. The CC_50_ and TI were 20.51 µm and 19.35, respectively. Molecular docking indicated that GOS likely targets the RdRp of all examined coronaviruses (Figure S12, Supporting Information), resulting in a pan‐coronavirus inhibitor for future coronavirus outbreaks.

## 3 Discussion

The COVID‐19 pandemic has caused great losses to global health and economic development. The emergence of fast‐spreading SARS‐CoV‐2 variants has exacerbated the threat to the world, although vaccines have been widely used worldwide. SARS‐CoV‐2 is not the last nasty coronavirus; some coronaviruses can spread among a wide variety of animal species and have the potential for cross‐species transmission.^[^ ^2^, ^3^ ^]^ Therefore, there is an urgent need to develop a broad‐spectrum anti‐coronavirus drug that is effective not only against SARS‐CoV‐2 but also against other coronaviruses that are potentially threatening in the future.

RdRp is an essential enzyme for viral replication and has emerged as an optimal target for the development of antiviral drugs.^[^ ^47^ ^]^ For different CoVs, the similarity of the amino acid sequence for viral RdRp ranges from 70% to 100%,^[^ ^18^ ^]^ suggesting that RdRp could act as an ideal target for wide‐ranging coronavirus inhibitors. NIs can imitate the natural substrates of CoV RdRp and result in fast or slow chain termination based on their geometry and binding affinity, making them one of the most promising RdRp inhibitors. Some RdRp NIs have been assessed to explore their broad‐spectrum efficiency against CoVs. Remdesivir is one of the most representative NIs and has been approved by the Food and Drug Administration (FDA) for COVID‐19 treatment. It occupies the central position of the catalytic active site, forms a covalent bond with the primer RNA strand, and blocks the replication of SARS, MERS and SARS‐CoV‐2 pathogens.^[^ ^18^, ^48^, ^49^ ^]^ Molnupiravir is another FDA‐approved drug for the treatment of COVID‐19 by targeting viral RdRp, which has shown a significant reduction in the rate of hospitalization or death of patients with COVID‐19.^[^ ^50^ ^]^ In addition to FDA‐approved NIs, some more effective, affordable, and convenient RdRp inhibitors have also been developed, such as GS‐441524, a major metabolite of remdesivir with broad‐spectrum antiviral activities across multiple virus families,^[^ ^23^ ^]^ and ATV006, a potent inhibitor of SARS‐CoV‐2 and its variants with high oral bioavailability.^[^ ^24^ ^]^ Although NIs have great potential in the prevention and control of coronaviruses, some limitations cannot be ignored. First, the replication proofreading function of 3′ to 5′ exoribonuclease (ExoN) in CoV genomes confers resistance to SARS‐CoV‐2 against many anti‐RdRp inhibitors, which has significantly hampered the development of NI‐based drugs against COVID‐19.^[^ ^51^ ^]^ Second, the effective dosage of some NIs needs to be adjusted to compete with highly concentrated intracellular natural nucleotide triphosphates (NTPs), which may increase the risk of drug toxicity.^[^ ^19^ ^]^ Additionally, the triphosphate forms of some NIs may be utilized by human DNA polymerases, leading to unwanted side effects.^[^ ^52^ ^]^

In contrast, NNIs exert antiviral activity by altering interactions between the enzyme substrate and the active core catalytic site of RdRp, which is expected to have fewer off‐target effects and better target specificity.^[^ ^53^ ^]^ To date, several RdRp NNIs have been developed for COVID‐19, including baicalein, corilagin and lycorine, most of these NNIs are plant‐derived natural products. Baicalein from *Scutellaria baicalensis* can block viral replication in cell culture systems by inhibiting the RdRp activity of SARS‐CoV‐2.^[^ ^27^ ^]^ It also exerts a broad‐spectrum inhibitory effect on the replication of several RNA viruses, including Zika virus and dengue virus.^[^ ^54^, ^55^ ^]^ Corilagin, acting as an NNI of SARS‐CoV‐2 RdRp, effectively inhibits polymerase activity in both cell‐free and cell‐based assays, which is likely to land at the palm domain of RdRp and prevents conformational changes required for nucleotide incorporation by RdRp.^[^ ^29^ ^]^ Lycorine, a bioactive pyrrolidine alkaloid isolated from the bulbs of *Lycoris radiata*, exhibits broad‐spectrum antiviral effects against several CoV pathogens,^[^ ^56^, ^57^ ^]^ including SARS‐CoV, MERS‐CoV and SARS‐CoV‐2. Although these plant‐derived NNI may be promising candidates for COVID‐19 management, their interaction modes with RdRp are not fully clear, their inhibitory activities against SARS‐CoV‐2 have not been verified in vivo, and their inhibition against other coronaviruses has not yet been tested in detail.

Natural product‐derived molecules have made great contributions to the discovery of effective RdRp inhibitors against a variety of viruses.^[^ ^58^, ^59^ ^]^ In this study, GOS produced by *Gossypium* plant was identified and characterized as a novel inhibitor of SARS‐CoV‐2 RdRp using virtual screening and biochemical approaches. GOS effectively suppressed the replication of SARS‐CoV‐2 and its variants in in vitro and in vivo assays. Combining cryo‐EM and gel shift assays, we showed that GOS binds to the active cavity of SARS‐CoV‐2 RdRp and prevents the binding of the primer‐template duplex, thus effectively inhibiting polymerase activity and displaying its antiviral effect. The function and structure of RdRps are conserved in ssRNA viruses,^[^ ^14^, ^15^ ^]^ especially coronaviruses (Figure 5a and Figure S11, Supporting Information). Multiple lines of evidence have revealed that GOS shows broad inhibitory activity against coronaviruses from SARS‐CoV‐2 and its variants to other coronaviruses, including PEDV, SADS‐CoV, IBV and PDCoV, indicating that GOS is a promising natural product for broad‐spectrum inhibition of coronaviruses and has great potential for use in the prevention and control of both known and unknown coronaviruses.

Drug repurposing is a promising strategy for the development of old antiviral agents as new therapeutics. GOS is a unique secondary metabolite of *Gossypium* plant that has a variety of pharmacological effects.^[^ ^60^, ^61^, ^62^ ^]^ In China, GOS is available as a prescription drug for the treatment of gynecological diseases^[^ ^63^ ^]^ and is under phase III clinical trial as an adjuvant drug for the treatment of advanced non‐small cell lung cancer with high apurinic/apyrimidinic endonuclease 1 (*APE1*) expression (NCT number: NCT01977209). Therefore, repurposing GOS for its characterization as a pan‐coronavirus inhibitor will provide an effective strategy for rapid response to outbreaks of other coronaviruses in the future. However, high concentrations of free GOS may be responsible for some side effects, including respiratory distress, anorexia, weakness and apathy.^[^ ^64^ ^]^ Therefore, it is need to explore the optimal dosages of GOS to achieve the best antiviral effect and the least side effects in preclinical models and ultimately in human clinical trials. Besides, to test and verify the in vivo anti‐SARS‐CoV‐2 activity of GOS under limited resources of Animal Biology Security Laboratory (ABSL) at the time, we chose the commonly used i.m. and i.n. administration route to deliver GOS in this study; other delivery methods, such as intravenous injection, intraperitoneal injection or oral administration, need to be further explored in future research. Finally, because of hindered rotation around the binaphthyl bond, GOS exists as (+) and (−) enantiomers. In this study, the antiviral effect of GOS was unaffected by its optical activity. Considering that cells are more sensitive to (−)‐GOS,^[^ ^65^ ^]^ it may be a good choice to develop antiviral drugs derived from (+)‐GOS in future research.

Since the genes associated with GOS biosynthesis pathway are well characterized,^[^ ^66^ ^]^ gene editing and synthetic biology approaches should be readily accessible to facilitate the production of GOS and its derivatives in cotton plant. GOS contains a parent nucleus with many active hydroxyl and aldehyde groups, which can be modified to develop new derivatives with stronger inhibitory effects on current and future CoV RdRps. Taken together, GOS is a promising scaffold for the development of veterinary drugs and medicines to block pathogenic coronaviruses in cross‐kingdom hosts.

## 4 Conclusions

Through virtual screening and experimental validation, we identified GOS as an effective SARS‐CoV‐2 RdRp inhibitor. Cryo‐EM and biochemical assays showed that GOS binds directly to the active cavity of SARS‐CoV‐2 RdRp, disturbing the binding of the RNA primer‐template duplex, thus inhibiting the replication of SARS‐CoV‐2 in vitro and in vivo. Based on the highly conserved coronavirus RdRps, we further found that GOS exhibited excellent antiviral activity against all examined viruses from the four main phylogenetic branches of coronaviruses. Taken together, GOS is a conventional drug but a novel broad‐spectrum anti‐coronavirus inhibitor that offers a potential treatment option for current COVID‐19 and future coronavirus infections.

## 5 Experimental Section

### Virtual Screening of Candidate Natural Inhibitors against RdRp of SARS‐CoV‐2

Based on in‐house generated database of cotton antiviral natural products containing 881 small molecules established in the authors’ laboratory, a virtual screening of anti‐SARS‐CoV‐2 natural products was performed using AutoDock Vina in PyRx0.8^[^ ^67^ ^]^ with a known RdRp inhibitor baicalein^[^ ^27^ ^]^ as a positive control. The structure of SARS‐CoV‐2 RdRp (PDB 7BV2) was used to generate the receptor grid for natural products’ docking simulations. The center of the active site of the grid was determined according to the position of RTP in the structure. The natural products with a binding energy lower than that of positive control had been selected for further analysis, with the sources of the chemicals listing in Table S4, Supporting Information.

### Constructs and Expression of the SARS‐CoV‐2 NSP12‐7‐8 Complex

The NSP12‐7‐8 complex (NSP12:NSP7:NSP8 = 1:1:2) was prepared according to a previous study.^[^ ^13^ ^]^ Following codon optimization and chemical synthesis, the full‐length NSP12 gene (YP_0 097 25307.1) was cloned into a modified pFastBac baculovirus expression vector containing a 5′ ATG start sequence, *C*‐terminus tobacco etch virus (TEV) protease sequence, and a His8 tag. The derivative recombinant plasmid contained NSP12 with additional methionine at the *N*‐terminus and GGSENLYFQGHHHHHHHH at the *C*‐terminus. Full‐length NSP7 (YP_0 097 42614.1) and NSP 8 (YP_0 097 25304.1) genes were cloned into the pFastBac vector containing a 5′ATG start sequence. All resulting plasmids were transformed into *Spodoptera frugiperda* (Sf9) cells at a ratio of 1:2:2 (NSP12: NSP7: NSP8) at a multiplicity of infection (MOI) of ≈5. The cells were harvested after 48 h of infection at 27 °C and cell pellets were stored at −80 °C.

Additionally, the synthesized full‐length NSP7 and NSP8 genes were cloned into a modified pET‐32a(+) vector with a 5′ATG start sequence at the *N*‐terminus, a TEV cleavage site, and a His8 tag at the *C*‐terminus. The plasmids were transformed into *Escherichia coli* BL21 (DE3) cells (Invitrogen). After the bacterial cultures grown at 37 °C to an optical density (OD) of 0.6, the gene expression was induced with the isopropyl ß‐D‐1‐thiogalactopyranoside (IPTG) of 0.1 mm and a temperature of 16 °C. The bacterial cultures were grown for additional 18–20 h, and then the independent cultures were compressed into pellets and stored at −80 °C.

### Purification of NSP12‐7‐8 Complex

The co‐expressed NSP12‐7‐8 complex was purified as previous study.^[^ ^68^ ^]^ Briefly, Sf9 cells expressing the NSP12‐7‐8 complex were resuspended and incubated for 20 min with stirring in binding buffer containing 25 mm HEPES (pH 7.4), 1 mm magnesium chloride, 300 mm sodium chloride, 1 mm tris (2‐carboxyethyl) phosphine (TCEP), 0.1% v/v IGEPALCA‐630 (Anatrace), 25 mm imidazole, and 10% v/v glycerol with an additional ethylenediaminetetraacetic acid (EDTA)‐free protease inhibitor cocktail (Bimake). Cells were lysed in a high‐pressure homogenizer at 500 bar and centrifuged at 3000 × *g* for 30 min. The supernatant was collected and incubated with nickel‐nitrilotriacetic acid (Ni‐NTA) beads (GE Healthcare) at 4 °C for 2 h, followed by washing with 20 column volumes of wash buffer (25 mm HEPES, pH 7.4, 1 mm TCEP, 1 mm magnesium chloride, 25 mm imidazole, 300 mm sodium chloride, and 10% v/v glycerol). Proteins were eluted with 3–4 column volumes of elution buffer (25 mm HEPES, pH 7.4, 300 mm sodium chloride, 300 mm imidazole, 1 mm magnesium chloride, 1 mm TCEP, and 10% v/v glycerol).

The purification of NSP7 and NSP8 expressed in bacteria was similar to that described in a previous study.^[^ ^69^ ^]^ Briefly, bacteria were lysed in a high‐pressure homogenizer at 800 bars. The lysate was centrifuged at 25 000 × *g* for 30 min to remove debris, and then incubated with Ni‐NTA beads (GE Healthcare). After washing with a buffer containing 50 mm imidazole, the proteins were eluted with a buffer containing 300 mm imidazole. The proteins were incubated with TEV protease overnight to remove the tag and concentrated using centrifugal filters with a molecular weight cutoff of 3 or 30 kDa (Millipore Corporation). The concentrated proteins were separated by size on a SuperdexS75 10/300 GL column in 25 mm HEPES (pH 7.4), 200 mm sodium chloride, and 5% v/v glycerol. The NSP7 and NSP8 fractions were collected and stored at −80 °C until further use.

The co‐expressed NSP12‐7‐8 complex was incubated with additional NSP7 and NSP8 expressed in bacteria at a molar ratio of 1:1:2 at 4 °C for 4 h. The incubated complex was then concentrated using a centrifugal filter with a 100 kDa molecular weight cut‐off (Millipore Corporation) and subjected to size separation using a SuperdexS200 10/300GL column in 25 mm HEPES (pH 7.4), 300 mm sodium chloride, 0.1 mm magnesium chloride, and 1 mm TCEP. Fractions containing monomer complexes were collected for subsequent research.

### Expression and Purification of SARS‐CoV‐2 Variants RdRp Complex

To obtain two types of mutant RdRp proteins, RdRp^P323L^ and RdRp^P323L; G671S^, the two full‐length NSP12 genes with mutants of P323L, P323L and G671S were chemically synthesized with codon optimization. Expression and purification of the two mutant NSP12‐7‐8 complexes were performed in the same way as described above.

### Gel‐Based Enzymatic Activity and Inhibition Assays of SARS‐CoV‐2 RdRp

The enzymatic activity of RdRp (including the original RdRp, RdRp^P323L^, and RdRp^P323L;G671S^) was tested using the gel‐based method described in a previous study.^[^ ^68^ ^]^ Briefly, to obtain poly A template‐primer RNA, a 30‐base poly‐A template (5′‐AAAAAAAAAAAUAACUUAAUCUCACAUAGC‐3′) and a 20‐base primer with the carboxyfluorescein (FAM) label at the 5′ (5′‐FAM‐GCUAUGUGAGAUUAAGUUAU‐3′) were mixed in a molar ratio of 1:1 in a buffer of 10 mm Tris‐HCl, pH 8.0, 25 mm NaCl and 2.5 mm EDTA. The mixture of template and primer RNA was denatured at 95 °C for 5 min and then cooled slowly to room temperature.

The RdRp complex (final concentration of 2.0 µm), 3.0 µm poly‐A primer‐template and 10 mm uridine 5′‐triphosphate (UTP) (Macklin) were incubated in a reaction buffer containing 20 mm Tris (pH 8.0), 10 mm KCl, 6 mm MgCl_2_, 0.01% Trition‐X100, 1.0 mm DTT and 1.15 U µL^−1^ RNase inhibitor at 37 °C for 60 min. Then, a quenching buffer of 94% formamide and 30 mm EDTA was added to the reaction system at a volume ratio of 2:1. The final reaction sample was mixed with 2× urea‐TBE loading buffer, followed by heating for 5 min at 95 °C. The elongated product and primer RNA were separated by loading the sample onto a 20% urea polyacrylamide gel electrophoresis (urea‐PAGE) denatured gel and were run for 2 h at 120 V. The RNA bands were imaged using a multifunctional fluorescence imager.

The inhibition assays of RdRp were similar to those of enzymatic activity assay, except for the addition of the candidate natural products and the incubation at 37 °C for 1 h before adding UTP. Preliminary screening for RdRp inhibitors was performed using 50 µm of each compound. Natural products that exhibited good inhibition of RdRp were diluted to different final concentrations to determine the half‐maximal inhibitory concentration (IC_50_).

### Fluorescence‐Based Inhibition Assays of SARS‐CoV‐2 RdRp

Fluorescence‐based inhibition assays of SARS‐CoV‐2 RdRp were performed using a 30‐base poly‐A template (5′‐AAAAAAAAAAAUAACUUAAUCUCACAUAGC‐3′) and a 20‐base primer (5′‐GCUAUGUGAGAUUAAGUUAU‐3′). The reaction for RNA extension was the same as that for the gel‐based assays, except for the detection method. After incubation with UTP, the reaction was stopped by the addition of 20 µL of 100 mm EDTA and then mixed with PicoGreen (1/200). Following incubation for 5 min, the fluorescence intensity was measured using a Tecan Spark 20 m multimode microplate reader at excitation and emission wavelengths of 485 and 530 nm, respectively.

### Gel Mobility Shift Assays

To reconfirm whether GOS inhibits RdRp activity by preventing RNA binding, a gel mobility shift assay was performed according to the reported methods.^[^ ^68^ ^]^ In the presence of GOS (0, 5, 20, 50, 100, 200, 500 and 1000 µm), 10 µg RdRp complex and 1.0 µg poly‐A template–primer RNA were incubated at room temperature for 30 min in the binding reaction containing 25 mm HEPES pH 7.4, 100 mm sodium chloride, 2 mm magnesium chloride, 1 mm DTT and 1.14 U µL^−1^ RNase inhibitor. The reaction product was then resolved on 4–20% native polyacrylamide gel running in 1× TBE buffer at 90 V for 50 min in a 4 °C cold room. The gel was imaged with a Tanon‐5200 Multi Fluorescence Imager according to the manufacturer's protocol.

### Cellular Anti‐SARS‐CoV‐2 Assay for Candidate Natural Products

All experiments with infectious SARS‐CoV‐2 were performed in the biosafety level 4 and animal biosafety level 4 facilities in the Harbin Veterinary Research Institute (HVRI) of the Chinese Academy of Agricultural Sciences (CAAS), which is approved for such use by the Ministry of Agriculture and Rural Affairs of China. Vero E6 cells (African green monkey kidney, ATCC) were maintained in Dulbecco's modified Eagle's medium (DMEM) containing 10% fetal bovine serum (FBS) and antibiotics and cultured at 37 °C in an incubator with 5% CO_2_. Cellular antiviral assays were performed following a previous method.^[^ ^44^ ^]^ For further screening of the natural SARS‐CoV‐2 inhibitors, Vero E6 cells were pre‐treated for 1 h with 50 µm natural products which were obtained from the above RdRp inhibition assays. Then the cells were infected with SARS‐CoV‐2/HRB26/human/2020/CHN (HRB26, GISAID access no. EPI_ISL_459 909) at an MOI of 0.005 for 1 h, cells were then washed with PBS and continued to be cultured for 24 h with a virus growth medium containing 50 µm natural products or vehicle solution. The virus titer in the cell culture supernatant was determined as the copies of viral genomic RNAs by qPCR. Briefly, vRNA of SARS‐CoV‐2 was extracted using a QIAamp vRNA Minikit (Qiagen, Hilden, Germany). After reverse transcription using HiScript II Q RT SuperMix (Vazyme, Nanjing, China) of the vRNA, qPCR was performed to quantitate the RNA copies of viral *N* gene with specific primers (Table S5, Supporting Information). Inhibition rate (%) = [1 − (vRNA copies of treatment group/vRNA copies of control group)] × 100%. After antiviral screening at the cellular level, GOS, GOSAc, (−)‐GOS and GOP were selected to test their detailed antiviral activities. Besides, a human airway epithelial cell Calu‐3 was also introduced to further evaluated anti‐SARS‐CoV‐2 ability of GOS at the cellular level using the same methods.

### Inhibition Assays of GOS against SARS‐CoV‐2 Delta Variant

After being pre‐treated with GOS for 1 h, the vero E6 cells were infected with delta variant SARS‐CoV‐2/SZTH12/human/2021/CHN (SZTH12, the Genome Warehouse in National Genomics Data Center access no. GWHBGBU01000000) for 1 h (MOI 0.005), followed by a continuing culture for 24 h with a fresh virus growth medium containing GOS. vRNA extraction and qPCR detection were performed using the same methods with that of HRB26.

### Inhibition Assay of GOS against Other Coronaviruses

Animal viruses of SADS‐CoV (GenBank accession No. MG557844.1), PEDV (GenBank accession No. KT323980), IBV (strain M41^[^ ^70^ ^]^), and PDCoV (GenBank accession No. KU981059.1) were kept in HVRI of CAAS. To study the antiviral effect of GOS on SADS‐CoV, PEDV and IBV, the Vero E6 cells were pre‐treated for 1 h with GOS and then infected with SADS‐CoV, PEDV or IBV at an MOI of 0.01. After continuous infection for 1 h, cells were washed with PBS for three times and cultured for additional 24 h with a virus growth medium containing GOS. The vRNA of SADS‐CoV, PEDV or IBV was extracted and reverse‐transcribed. Finally, qPCR was performed with specific primers for SADS‐CoV and PEDV (Table S5, Supporting Information). Viral replication of IBV was determined by the amplification of the N gene with qPCR.^[^ ^70^ ^]^ The antiviral assay of PDCoV was similar with that of SADS‐CoV and PEDV, except that the cells used were swine testis (ST) cells.

### Cell Viability Assays

The viability of cells was determined using a Cell Titer‐Glo kit (Promega, Madison, WI, USA) following the manufacturer's instructions. Vero E6 cells were seeded in an opaque 96‐well plate and cultured at 37 °C for 12 h. Cells were treated with GOS, GOSAc and (−)‐GOS at final concentrations of 0–10 µm or gossypetin at final concentrations of 0–60 µm and cultured for an additional 24 h. Finally, cell Titer‐Glo reagent was added to each well, and luminescence was measured with a GloMax 96 Microplate Reader (Promega, Madison, WI, USA). The viabilities of Calu‐3 and ST cells were tested using the same method with GOS concentrations of 0–25 µm.

### Anti‐SARS‐CoV‐2 Assays of GOS In Vivo

The antiviral effects of GOS were assessed in a SARS‐CoV‐2 mouse infection model as described in previous studies.^[^ ^44^ ^]^ Briefly, 4‐6‐week‐old female BALB/c mice (Vital River Laboratory Animal Technologies, Beijing, China) were randomly divided into three groups. Alternatively, mice were treated i.n. or i.m. with a loading dose of 50 (high‐dose) or 10 mg kg^−1^ (low‐dose) GOS followed by a daily maintenance dose of 25 (high‐dose) or 5 mg kg^−1^ (low‐dose). As controls, mice in the other two groups were treated with a vehicle solution (48% PEG300, pH 7.0). One hour after administration of the loading dose of GOS or vehicle solution, each mouse was inoculated i.n. with 10^3.6^ plaque forming units (PFUs) (50 µL) of HRB26M. On day 3 p.i., turbinates and lungs were collected for virus detection by qPCR and plaque forming units (PFUs) assays according to previously described methods.^[^ ^71^ ^]^ The mouse experiments were carried out in strict accordance with the recommendations of the Guide for the Care and Use of Laboratory Animals of the Ministry of Science and Technology of the People's Republic of China. The protocols were approved by the Committee on the Ethics of Animal Experiments of the HVRI of CAAS (Approval number 2020‐01‐01JiPi).

### Tissue Distribution of GOS Under Different Administration Routes

To understand the antiviral effects of GOS in SARS‐CoV‐2 mouse infection models, the distribution of GOS in lung and nasal tissues at different administration routes and times were studied. The administration routes and dosages were the same as those used in the anti‐SARS‐CoV‐2 assay in vivo. After 1 hpi and 3 dpi, the turbinates and lungs were collected, frozen, and sonicated at 4 °C for 30 min. The tissue extracts were filtered and used to evaluate the GOS content through ultra‐performance liquid chromatography‐electrospray ionization triple‐quadruple tandem mass spectrometry (UPLC‐ESI‐MS‐MS) according to a previously described method.^[^ ^72^ ^]^

### Cryo‐EM Sample Preparation and Data Acquisition

For subsequent EM analyses, NSP12‐7‐8 complex (0.8 mg mL^−1^) was incubated with GOS at a 1:10 molar ratio at 4 °C for 1 h. A 4 µL aliquot of GOS‐bound complex was applied to a glow‐discharged 300 mesh grid (Quantifoil Au R1.2/1.3) supported with a thin layer of reduced graphene oxide, blotted with filter paper for 3.0 s and plunge‐frozen in liquid ethane using an FEI Vitrobot Mark IV. Cryo‐EM micrographs were collected on a 300 kV Titan Krios (G3i) microscope (FEI) equipped with a K3 direct detection camera and a Gatan image filter (GIF: slit width of 20 eV). The micrographs were collected at a calibrated magnification of ×130 000, yielding a pixel size of 0.27 Å in super‐resolution mode. In total, 10 683 movies were collected at an accumulated electron dose of 50e^−^Å^−2^ s^−1^ on each micrograph, which was fractionated into a stack of 32 frames with a defocus range of −1.0 to −2.0 µm.

### Cryo‐EM Image Processing

Beam‐induced motion correction was performed on a stack of frames using MotionCorr2.^[^ ^73^ ^]^ Contrast transfer function (CTF) parameters were determined using CTFFIND4.^[^ ^74^ ^]^ A total of 10 108 good micrographs were selected for further data processing using Relion 3.1.^[^ ^75^ ^]^ Particles were auto‐picked using the auto‐picking program in Relion, followed by two rounds of reference‐free 2D classification. Next, 1 884 236 particles were selected from good 2D classes and subjected to two rounds of 3D classification using reconstruction of the apo‐RdRp complex (EMD 30 209) as a starting model. Two converged 3D classes with a feature containing one NSP12 and one NSP7 and two fragments of NSP8 were selected for the final round of 3D refinement. In the 3D classes, 1 224 147 particles from a 3D class showing the highest resolution feature with an additional density were selected for another round of 3D refinement, yielding a final reconstruction at a global resolution of 3.36 Å based on the gold‐standard Fourier shell correlation criterion of FSC = 0.143. In addition, 76 186 particles from a 3D class without additional density were refined to a resolution of 4.5 Å. The local resolution was then calculated for the highest‐resolution 3D map.

In addition, a focus classification approach that applied a mask around the region of additional density was performed for the 3.36 Å reconstruction, in which 33% of the particles were found with the binding of additional density.

### Cryo‐EM Model Construction

The model of the RdRp‐GOS complex was built by fitting the model of the apo structure of RdRp (PDB 7BV1) into the density map using UCSF Chimera,^[^ ^76^ ^]^ followed by manual model construction of GOS molecules in COOT^[^ ^77^ ^]^ and real‐space refinement in PHENIX.^[^ ^78^ ^]^ The model statistics are presented in Table S6, Supporting Information.

### Phylogenetic Analysis of Coronavirus RdRp

The CoV RdRp sequences were obtained from the GeneBank with the accession numbers list in Table S7, Supporting Information. Multiple alignment analysis was performed in DNAman. Evolutionary analyses were conducted in MEGA7^[^ ^79^ ^]^ with neighbor‐join method.

### Interactions between GOS and RdRp of Various Coronaviruses

RdRp structures of PEDV, SADS‐CoV, IBV, and PDCoV were predicted by Alphafold2 based on the corresponding sequence obtained from the GeneBank with the accession numbers list in Table S7, Supporting Information. The interaction of GOS with RdRps was predicted by AutoDock and visualized by PyMOL, ChimeraX, and Proteins*Plus*.^[^ ^80^ ^]^

### Statistical Analysis

All statistical analysis was performed using GraphPad Prism 9 and the statistic tests are described in the indicated figure legends. Nonlinear regression curve fitting was performed to calculate the EC_50_ and IC_50_ values.

## Conflict of Interest

The authors declare no conflict of interest.

## Author Contributions

W.W., W.L., Z.W., C.W., and W.L. contributed equally to this work. Conceptualization: F.L., M.R., and W.W.; experiment design: F.L., M.R., W.W., L.Q., and Z.B.; data curation and formal analysis: W.W., W.L., Z.W., C.W., W.L., Y.Z., J.L., T.D., L.S., and G.Z.; writing – original draft: F.L., M.R. and W.W.; writing – review & editing M.R., W.W., L.Q., Z.B., and Z.W.

## Supporting information

Supporting Information

Supplemental Table 1

Supplemental Table 2

Supplemental Table 3

Supplemental Table 4

## Data Availability

The data that support the findings of this study are available from the corresponding author upon reasonable request.
