# Supplementary material for: Gossypol Broadly Inhibits Coronaviruses by Targeting RNA‐Dependent RNA Polymerases
Source: Adv Sci (Weinh). 2022 Oct 20;9(35):2203499. doi: 10.1002/advs.202203499 (PMC9762316; doi:10.1002/advs.202203499)
Supplement: Supplementary file 1 — Supporting Information [file ADVS-9-2203499-s001.pdf]

ADVANCED SCIENCE
Open Access

Supporting Information

for *Adv. Sci.*, DOI 10.1002/advs.202203499

Gossypol Broadly Inhibits Coronaviruses by Targeting RNA-Dependent RNA Polymerases

Wenjing Wang, Wenkang Li, Zhiyuan Wen, Chong Wang, Weilong Liu, Yufang Zhang, Juncheng Liu, Tianze Ding, Lei Shuai, Gongxun Zhong, Zhigao Bu, Lingbo Qu*, Maozhi Ren* and Fuguang Li**

Supporting Information

Gossypol broadly inhibits coronaviruses by targeting RNA-dependent RNA polymerases

Wenjing Wang[a,c,e,†], Wenkang Li[a,c,†], Zhiyuan Wen[b,†], Chong Wang[b,†], Weilong Liu[f,†], Yufang Zhang[a,c], Juncheng Liu[a,c], Tianze Ding[a,c], Lei Shuai[b], Gongxun Zhong[b], Zhigao Bu[b,], Lingbo Qu[a,]*, Maozhi Ren[a,c,d,e,]*, Fuguang Li[a,c,e,]**

**Corresponding authors: Fuguang Li (E-mail: aylifug@caas.cn), Maozhi Ren (renmaozhi01@caas.cn), Lingbo Qu (qlb@zzu.edu.cn) and Zhigao Bu (buzhigao@caas.cn)*

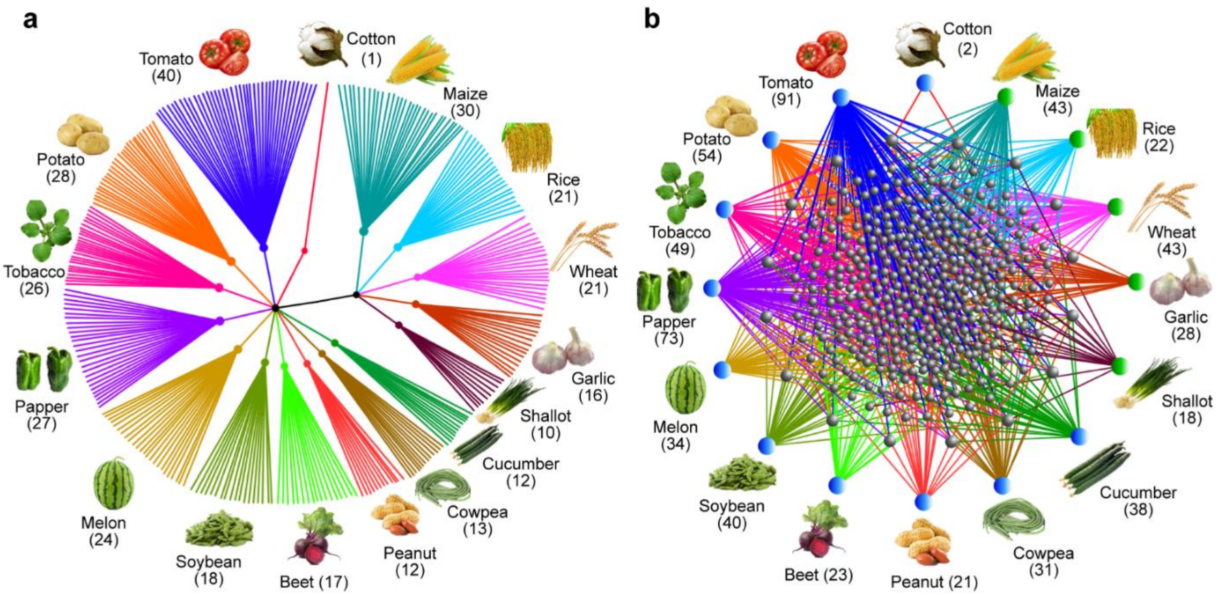

Figure S1. Statistical analyses of the interaction between host crops and pathogenic ssRNA viruses. a) The corresponding relationship between major host crops and pathogenic ssRNA viruses named after the related host crops. Each line represents a virus, and a different color corresponds to a different crop. b) The corresponding relationship between host crops and pathogenic ssRNA viruses. The colored spheres correspond to different crops, the gray spheres represent different ssRNA viruses, and the colored lines in the figure represent the relationship between host crops and ssRNA viruses. Virus information were obtained from the International Committee on Virus Taxonomy (ICTV) and NCBI virus database. Detailed hosts and viruses information are shown in Table S1. The statistics are as of December 31, 2021.

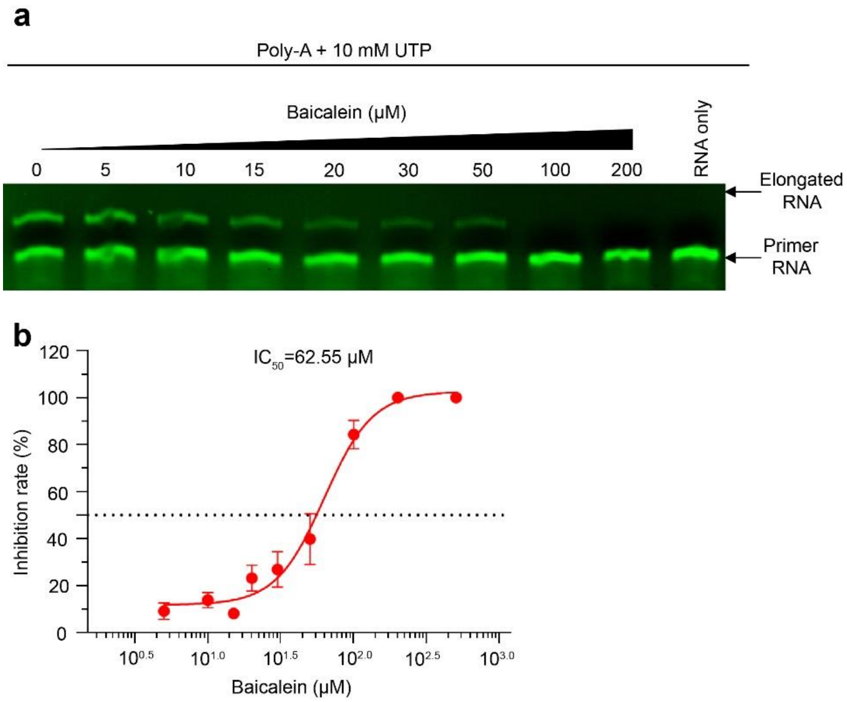

Figure S2. Inhibition of SARS-CoV-2 RdRp by baicalein. a) Gel-based assays of the elongation of partial RNA duplexes by the purified SARS-COV-2 RdRp and its inhibition by baicalein. b) The inhibition curves for baicalein obtained by fluorescence-based assays. Data are presented as mean ± s.d. (n=3).

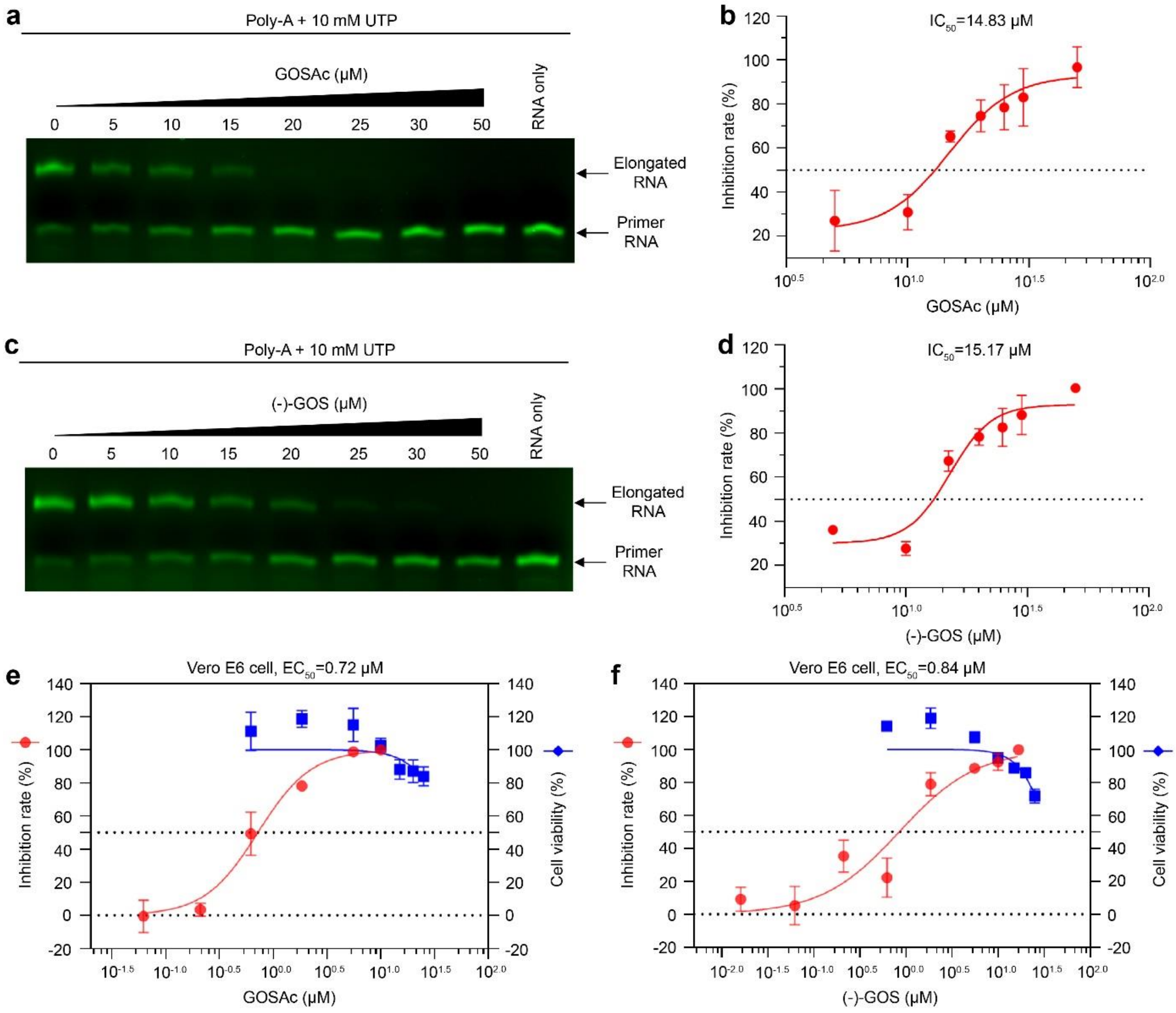

Figure S3. Inhibition of SARS-CoV-2 RdRp and SARS-CoV-2 by GOSAc and (-)-GOS. a and c) Gel-based assays of the elongation of partial RNA duplexes by the purified SARS-COV-2 RdRp and its inhibition by GOSAc (a) and (-)-GOS (c). b and d) The inhibition curves for GOSAc (b) and (-)-GOS (d) obtained by fluorescence-based assays. e and f) The inhibitory effects of GOSAc (e) and (-)-GOS (f) towards SARS-CoV-2 in Vero E6 cells, and assessment of potential cytotoxicity. The X-axis represents the working concentration of the natural product. The left and right Y-axes represent the inhibition rate and cell viability, respectively. Data are presented as mean ± s.d. (n=3).

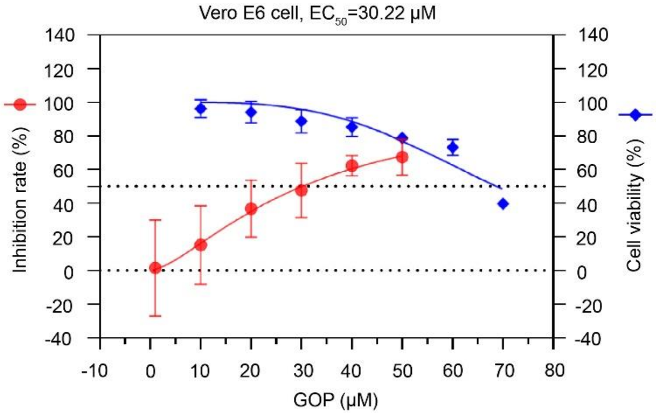

Figure S4. Inhibition of SARS-CoV-2 by GOP in Vero E6 cells. The X-axis represents the working concentration of GOP. The left and right Y-axes represent the inhibition rate and cell viability, respectively. Data are presented as mean ± s.d. (n=3).

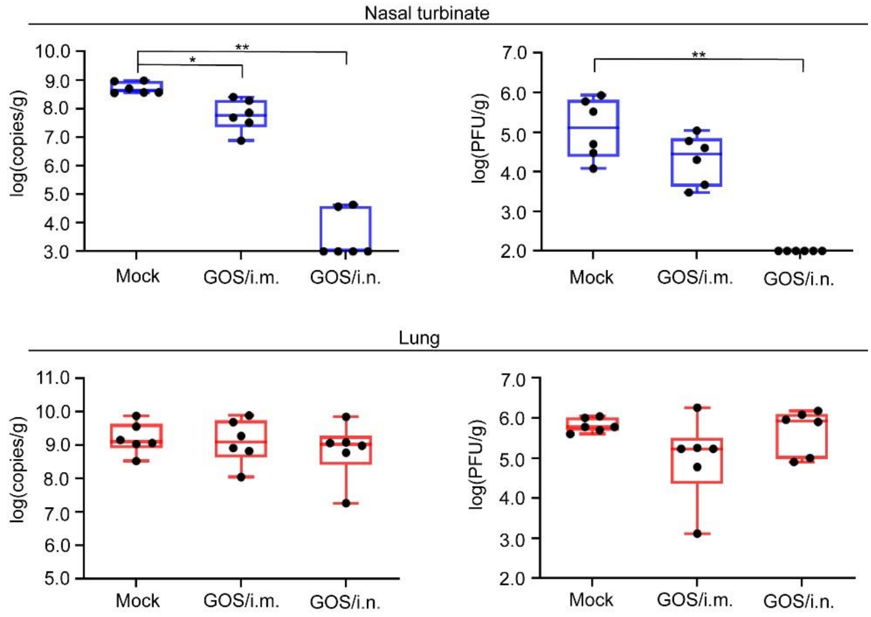

Figure S5. Antiviral effect of low-dose GOS *in vivo*. The viral RNA copies and infectious titres were detected by qPCR and viral titration. *i.m.* represents for intramuscular treatment; *i.n.* represents for intranasal treatment. Data are presented as mean ± s.d. (n=6). ** $p<0.01$, * $p<0.05$.

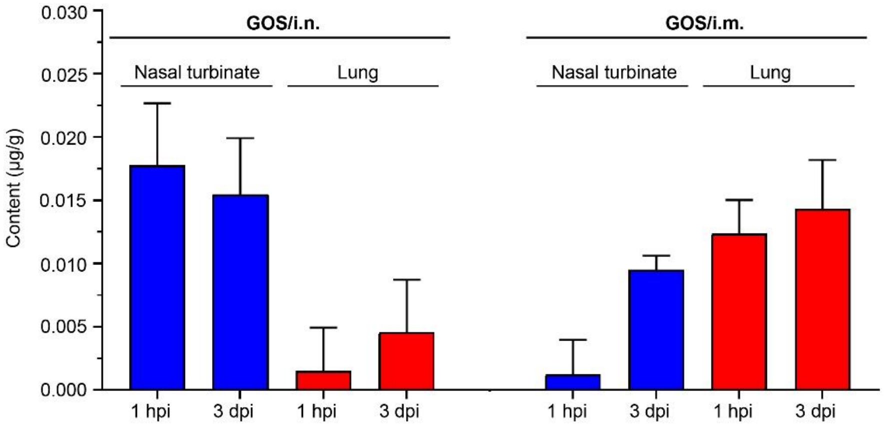

Figure S6. GOS distributions of GOS in nasal turbinates and lungs under different administration routes. The *i.m.* represents for intramuscular treatment; *i.n.* represents for intranasal treatment. Data are presented as mean ± s.d. (n=5).

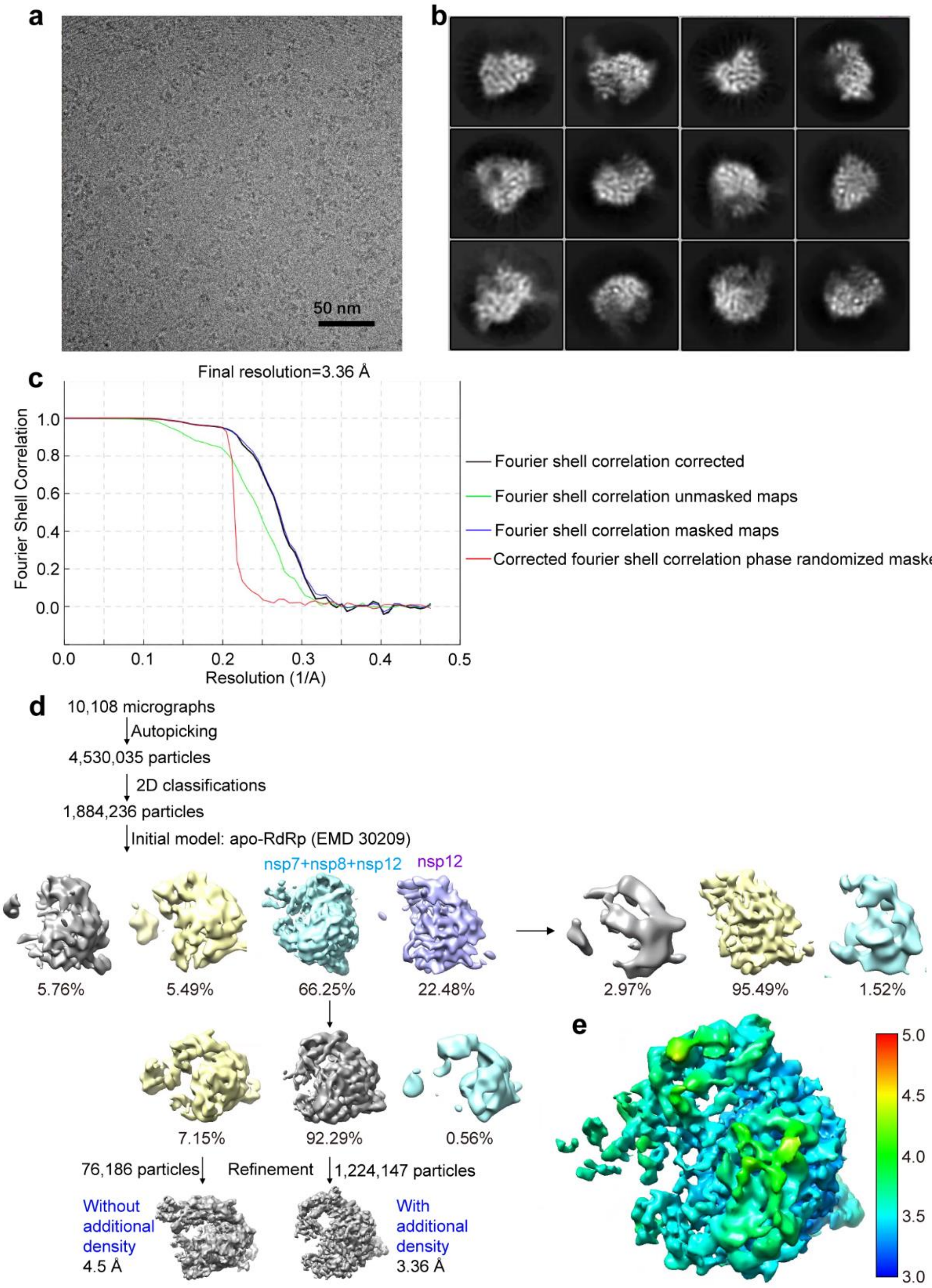

Figure S7. Single particle cryo-EM analysis of the SARS-CoV-2 RdRp-GOS complex. a) Representative cryo-EM image of the SARS-CoV-2 RdRp-GOS complex. b) Representative 2D class average of the SARS-CoV-2 RdRp-GOS complex. c) Fourier shell correlation curves for the cyo-EM map of the SARS-CoV-2 RdRp-GOS complex. d) Flowchart for cryo-EM work of the SARS-CoV-2 RdRp-GOS complex. e) Cryo-EM map of the SARS-CoV-2 RdRp-GOS complex with local colouration (coloured by local resolution (Å)).

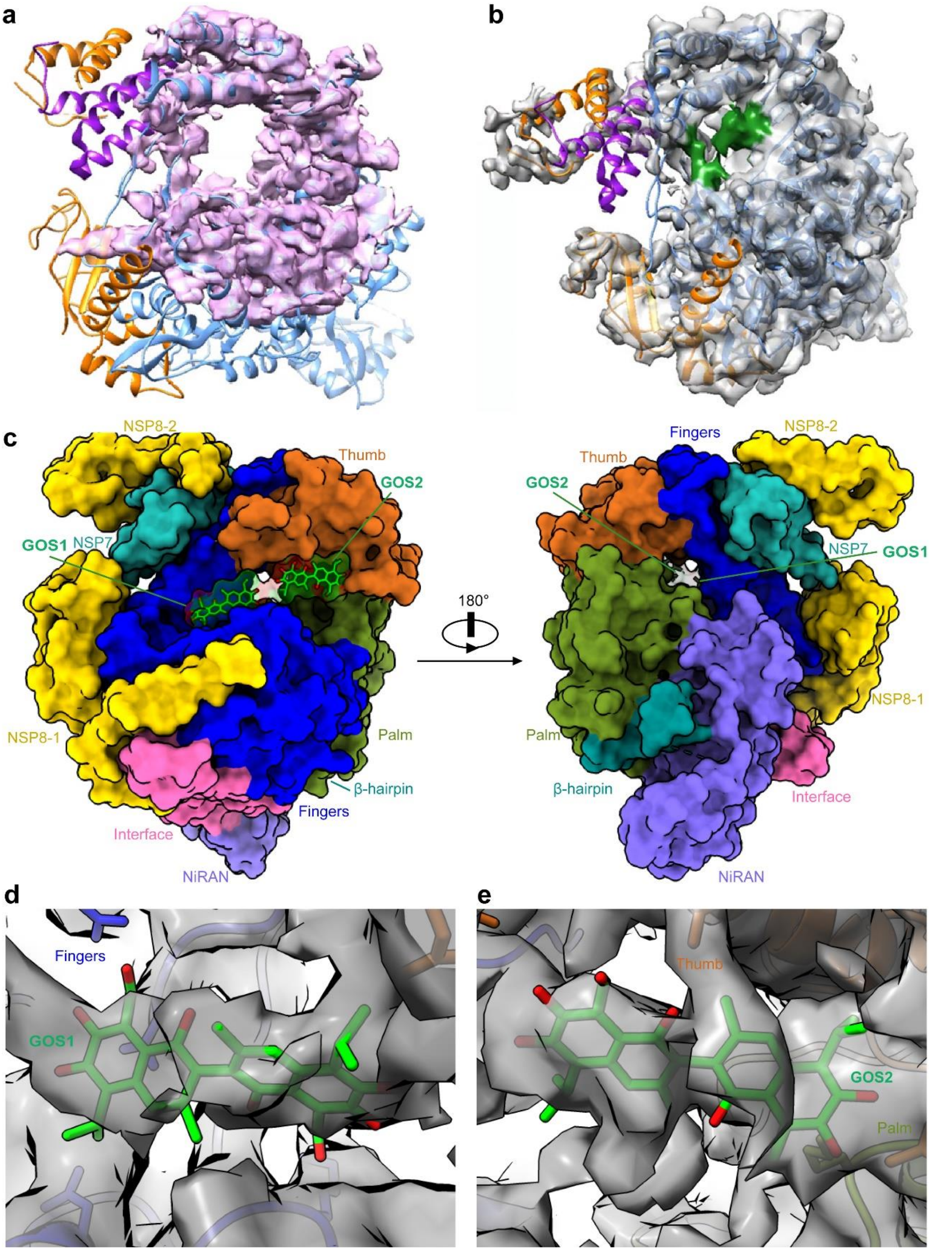

Figure S8. Cryo-EM reconstructions of SARS-CoV-2 RdRp-GOS complex. a) The map of apo structure of SARS-CoV-2 RdRp. b) The map of apo structure of SARS-CoV-2 RdRp-GOS. The zone colored with green was the additional density compared to the apo structure. c) Two surface views of SARS-CoV-2 RdRp-GOS complex. GOS was colored with green. d and e) EM maps for the two GOS molecules: GOS1 (d) and GOS2 (e).

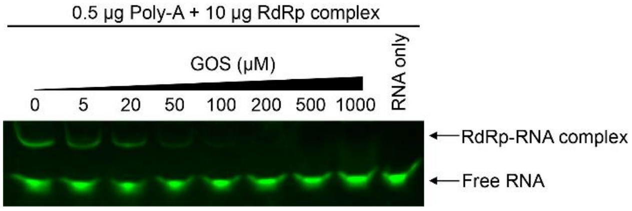

Figure S9. Gel mobility shift of the RdRp-RNA complex and the effect of GOS.

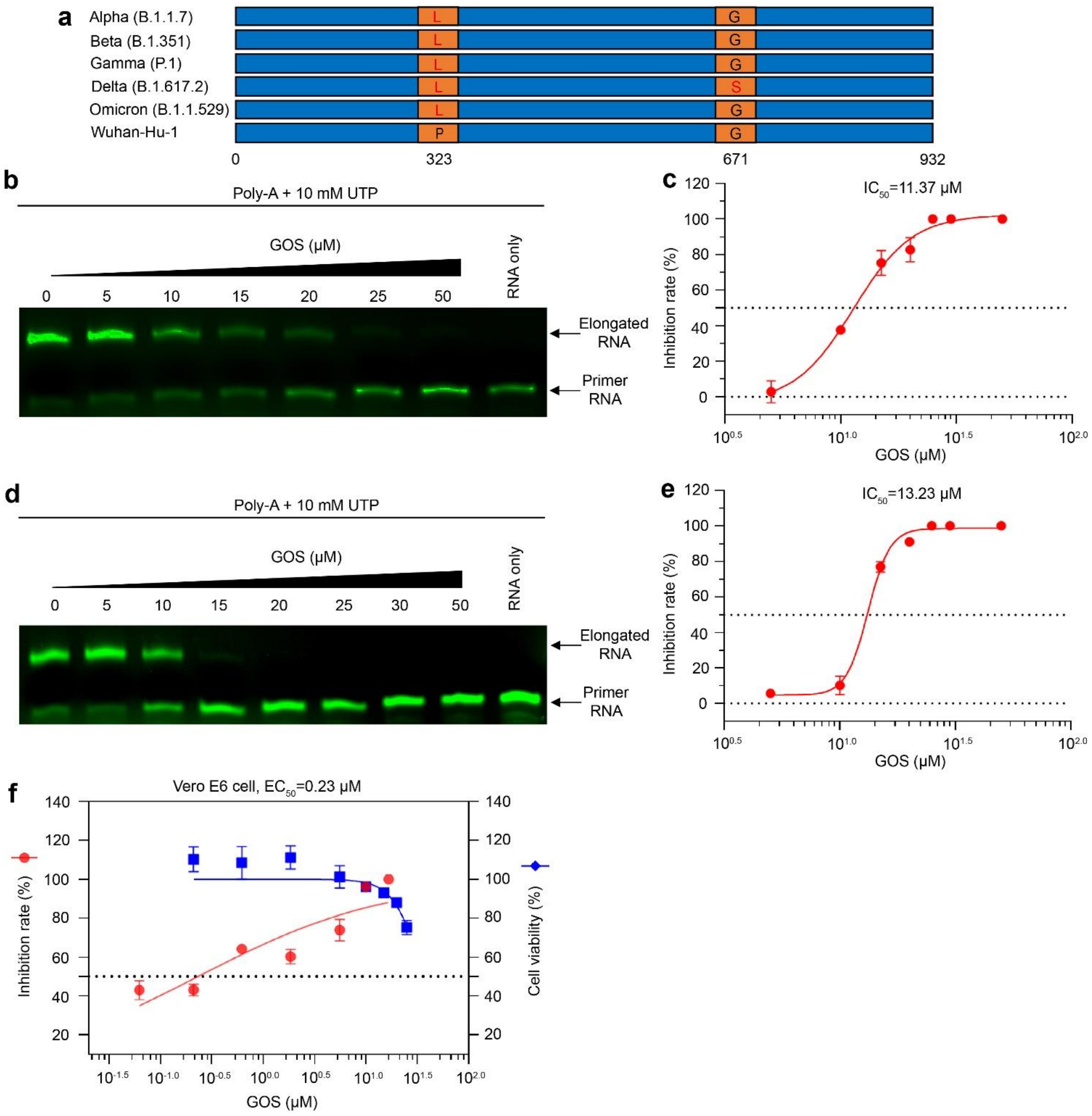

Figure S10. Inhibition effects of GOS on SARS-CoV-2 variant and mutated RdRp. a) RdRp mutations analysis of the six SARS-CoV-2 strains, in which the red letters represent mutations. b and c) Inhibitory effects of GOS on mutant RdRp^{P323L} obtained by gel-based (b) and fluorescence-based (c) assays. d and e), Inhibition effects of GOS on mutant RdRp$^{P323L;G671S}$ obtained by gel-based (d) and fluorescence-based (e) assays. The data represents the mean and standard deviation of three repeated measurements. (f) Inhibitory effects of GOS on SARS-CoV-2 delta variant. Data are presented as mean ± s.d. (n=3).

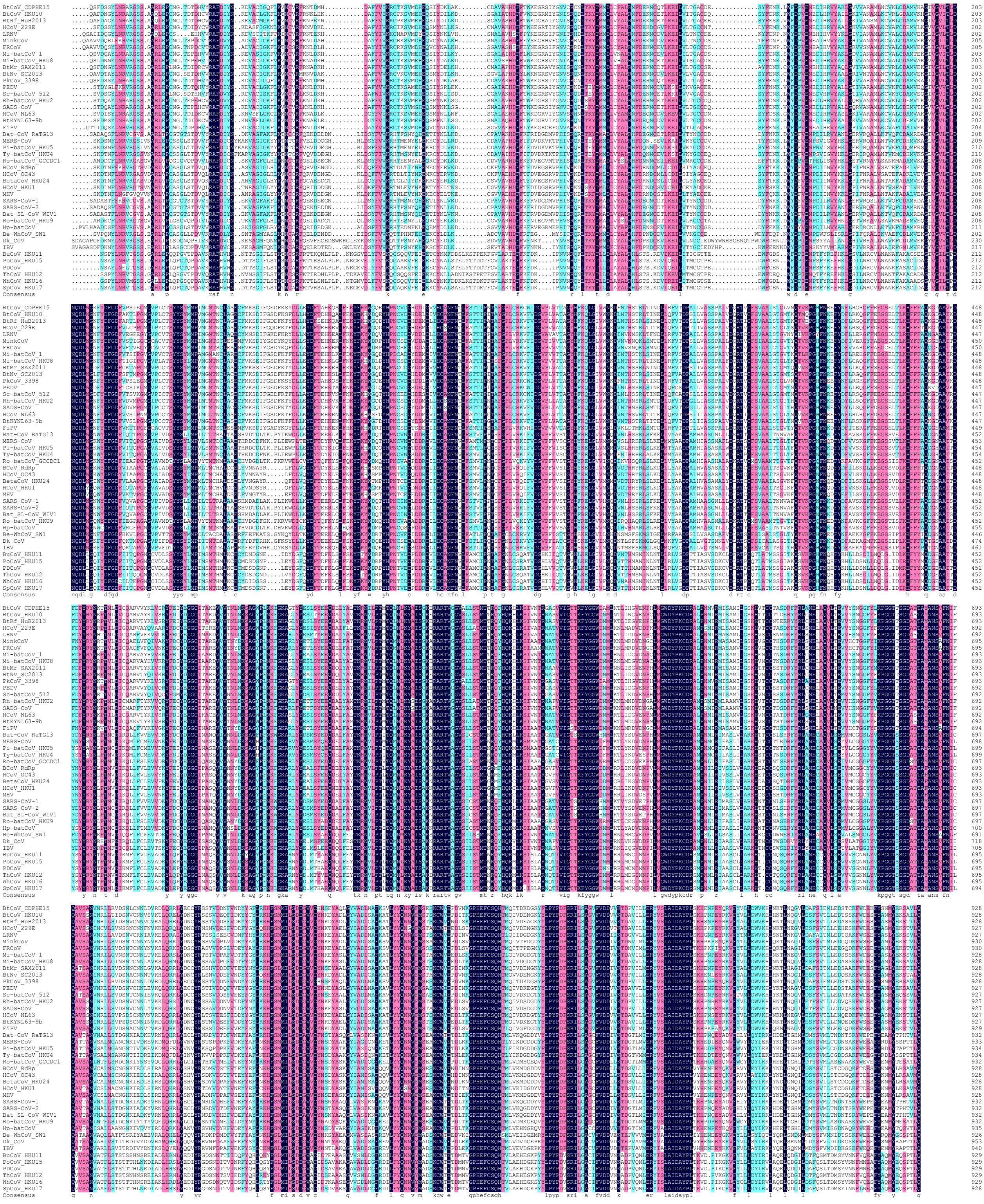

Figure S11. Multiple alignment analysis of CoV RdRp sequences. The GeneBank accession numbers of the RdRp sequences were list in Table S7. Colors mean the high conserved of the sequences.

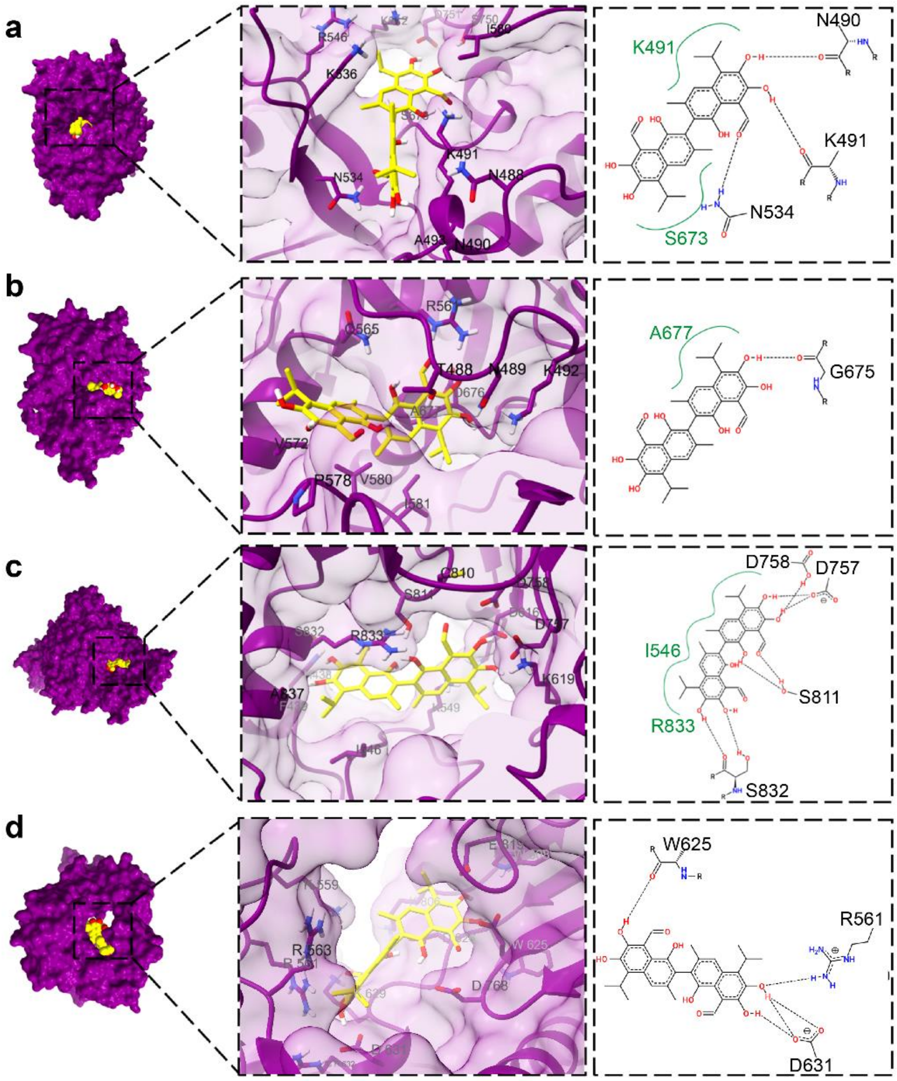

Figure S12. The binding poses of GOS with RdRps of representative coronaviruses. a to d), The surface view (left), cartoon view (middle) and interaction analysis (right) of GOS with RdRps of PEDV (a), SADS-CoV (b), IBV (c) and PDCoV (d). The predicted binding energies were -7.6 kcal/mol, -8.4 kcal/mol, -7.9 kcal/mol and -8.8 kcal/mol, respectively. Proteins are colored with purple, GOS is colored with yellow. The hydrogen bond is displayed as a black dashed line. The hydrophobicity is displayed as a green curve.

Table S3. Antiviral ability of candidate natural products towards SARS-CoV-2 in Vero E6 cells (n=3)[a].

Natural products	Inhibition rate towards SARS-CoV-2 (%)[b]
GOS	100.00
GOSAc	100.00
(-)-GOS	100.00
GOP	93.24
Baicalein	86.51
Gossypin	10.87

[a]Antiviral screening used 50 μM each natural product to research their antiviral ability towards SARS-CoV-2 at cellar level.

[b]Inhibition rate (%) = [1 – (viral RNA copies of treatment group / viral RNA copies of control group)] × 100%.

Table S5. Specific primers used in qPCR

Note	Primer Name	Sequence(5’-3’)
For SARS-CoV-2 N gene detection	SARS-CoV-2 N F	GGGGAACTTCTCCTGCTAGAAT
	SARS-CoV-2 N R	CAGACATTTTGCTCTCAAGCTG
	Fluorescent probe	FAM-TTGCTGCTGCTTGACAGATT-TAMRA
For PEDV detection	PEDV F	TTGGCATTTCTACTACCTC
	PEDV R	AGACGCCTTTCTGACACC
For SADS-CoV N gene detection	SADS-CoV N F	CCCCTAAACCGGCTCGTAA
	SADS-CoV N R	CAGAATTAGGAACACGCTTCCA
For PDCoV detection	PDCoV F	AGCAACCACTCGTGTTACTTG
	PDCoV R	CAACTCTGAAACCTTGAGCTG

Table S6. Cryo-EM data collection, refinement, and validation statistics

	SARS-CoV-2 RdRp-GOS<br>(EMD-34370)<br>(PDB 8GY6)
**Data collection and processing**	
Magnification	130k
Voltage (kV)	300
Electron exposure (e–/Å^2)	50
Defocus range (μm)	1.0-2.0
Pixel size (Å)	0.54
Symmetry imposed	C1
Final particle images (no.)	1,224,147
Map resolution (Å)	3.36
FSC threshold	0.143
**Refinement**	
Initial model used (PDB code)	7BV1
Map sharpening *B* factor (Å^2)	-100
R.m.s. deviations	
Bond lengths (Å)	0.007
Bond angles (°)	0.723
Clashscore	33.36
Ramachandran plot	
Favored (%)	83.44
Allowed (%)	16.56
Outliers (%)	0.00

Caption for Table S1. (separate file): List of host crops and the corresponding pathogenic ssRNA viruses.

Caption for Table S2. (separate file): List of cotton natural products and their binding energies with SARS-CoV-2 RdRp.

Caption for Table S4. (separate file): List of the chemicals sources.

Caption for Table S7. (separate file): The GeneBank accession numbers of CoV RdRps.
